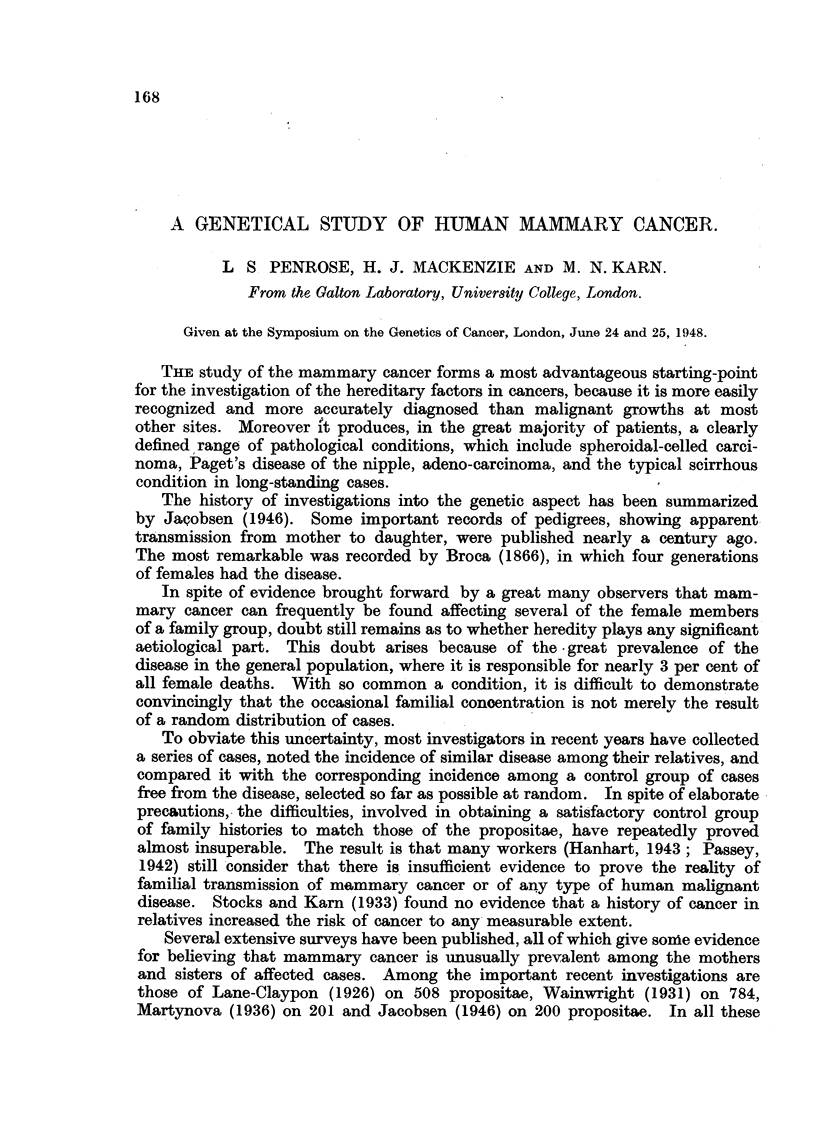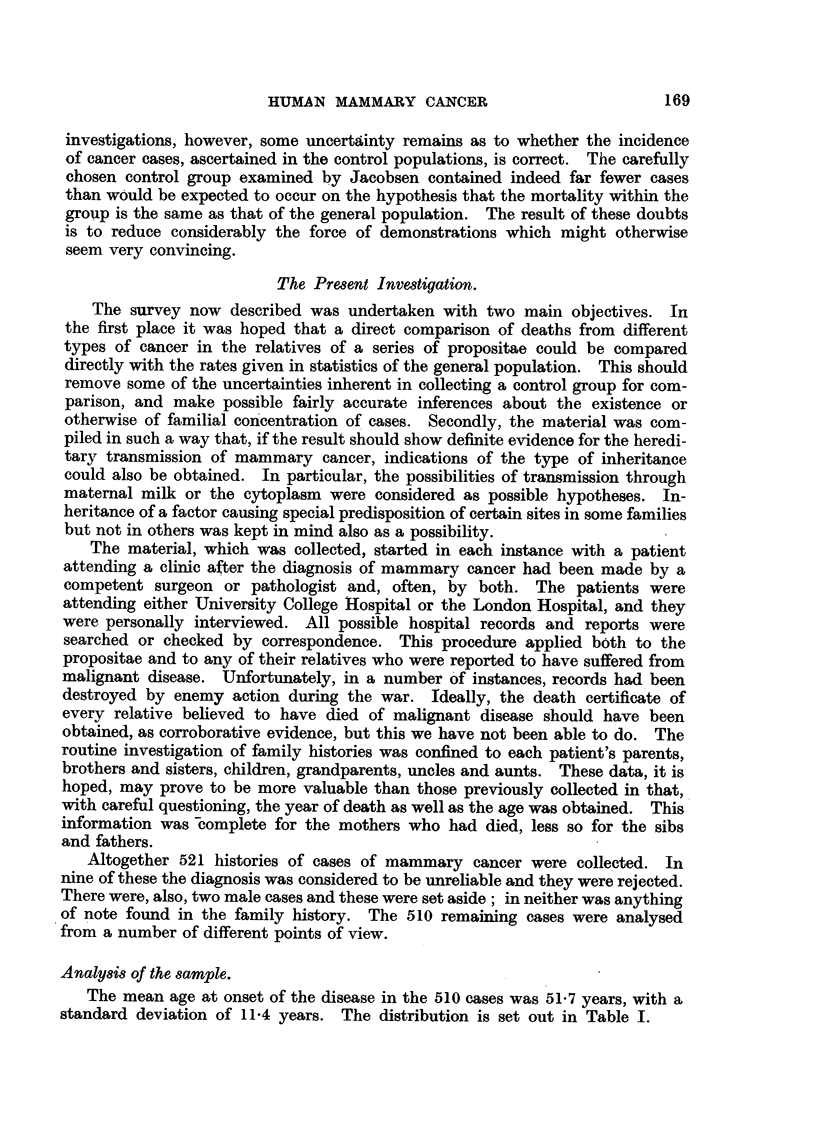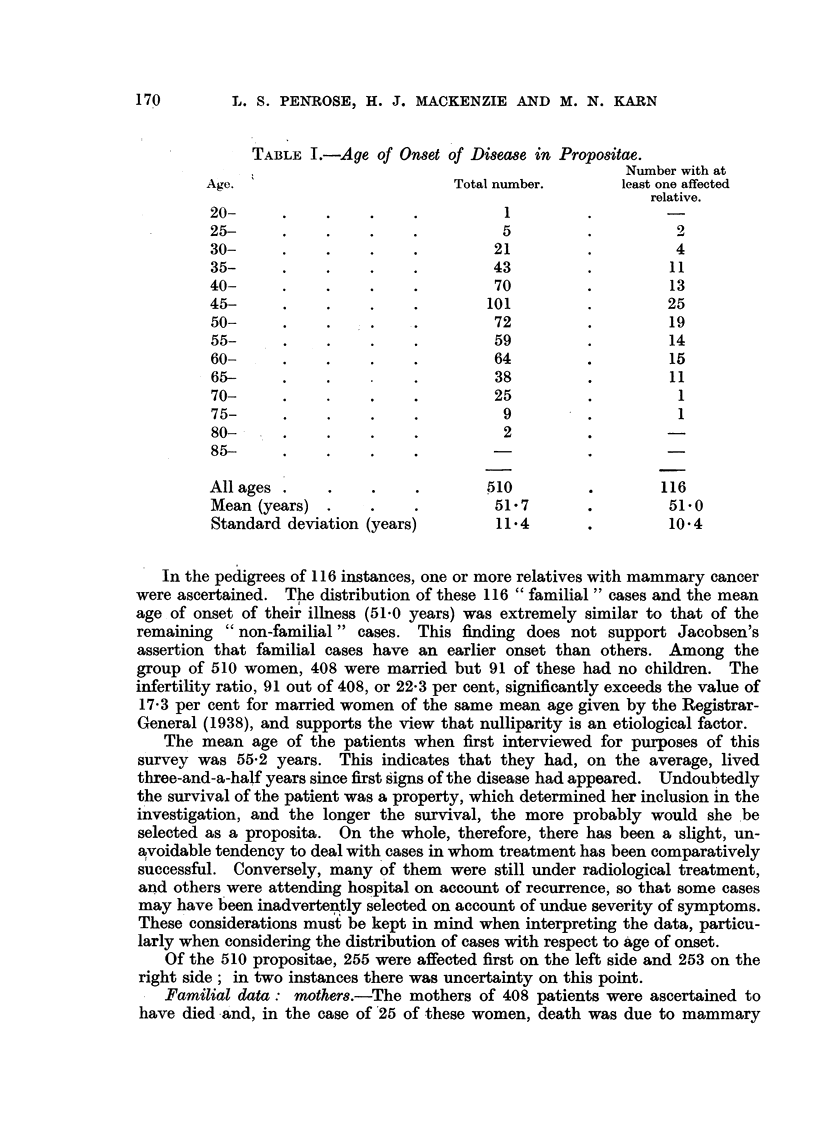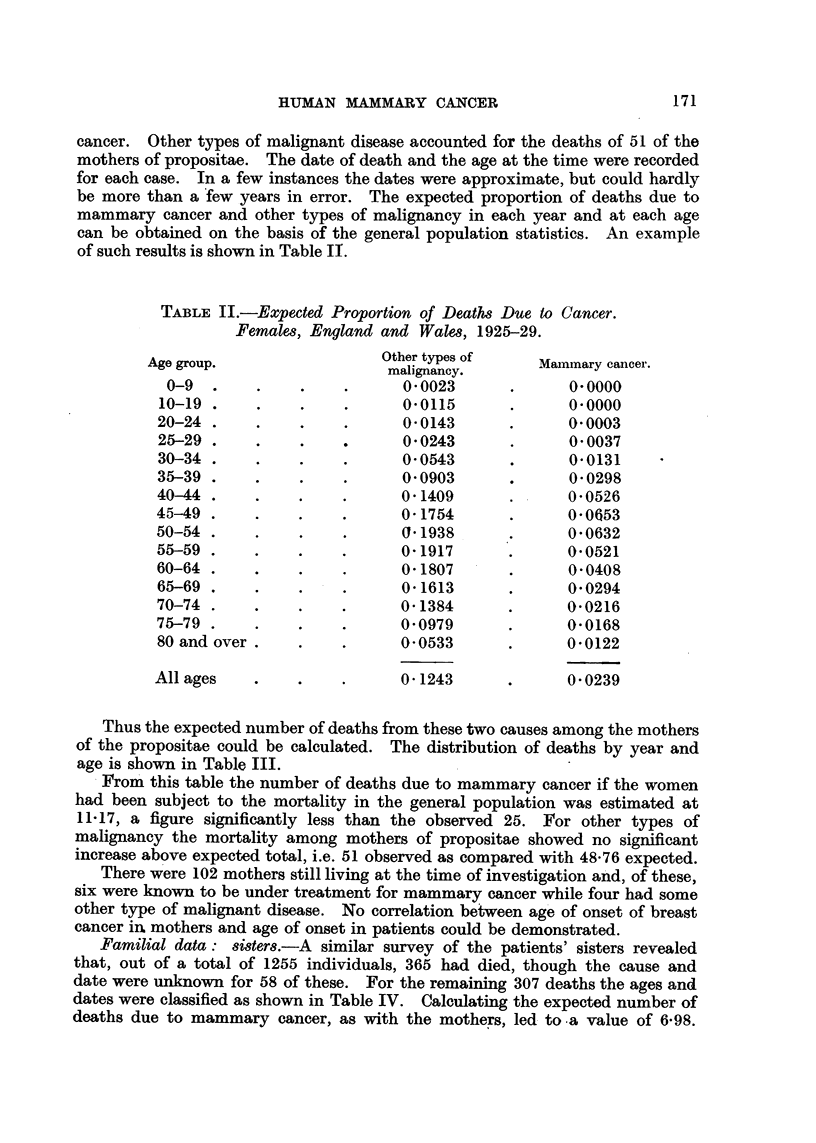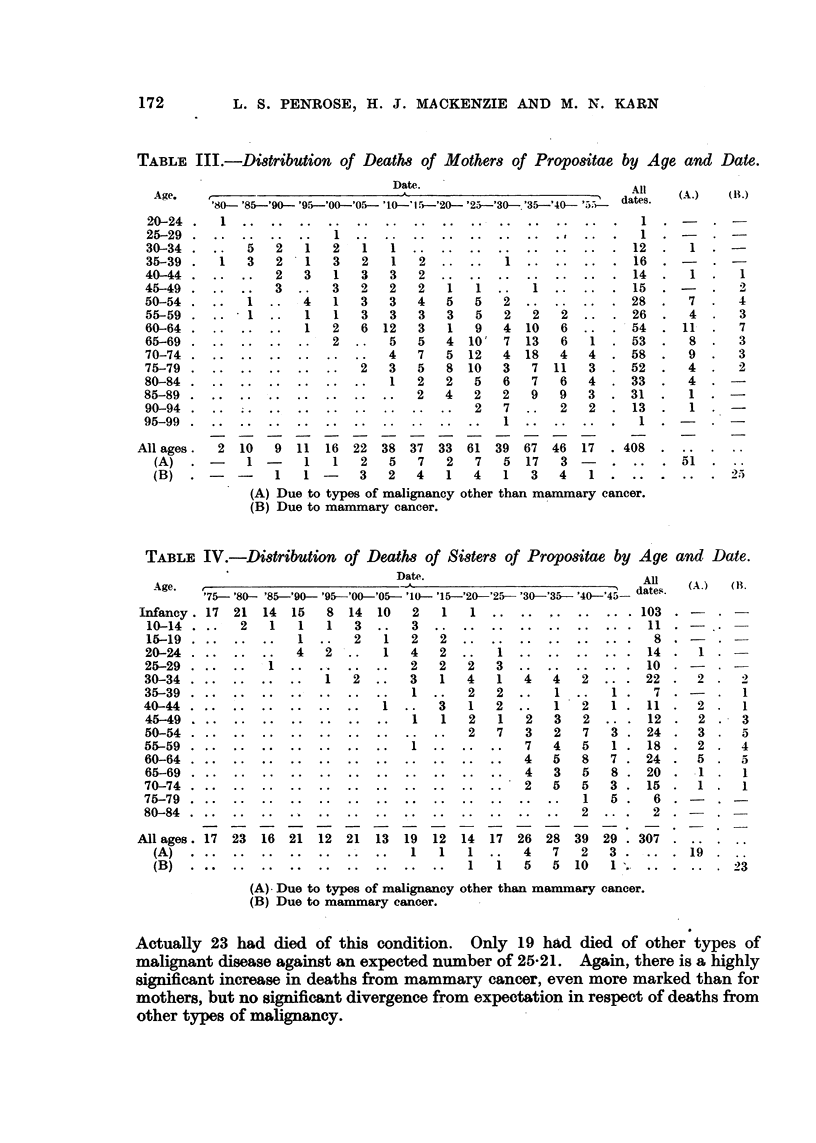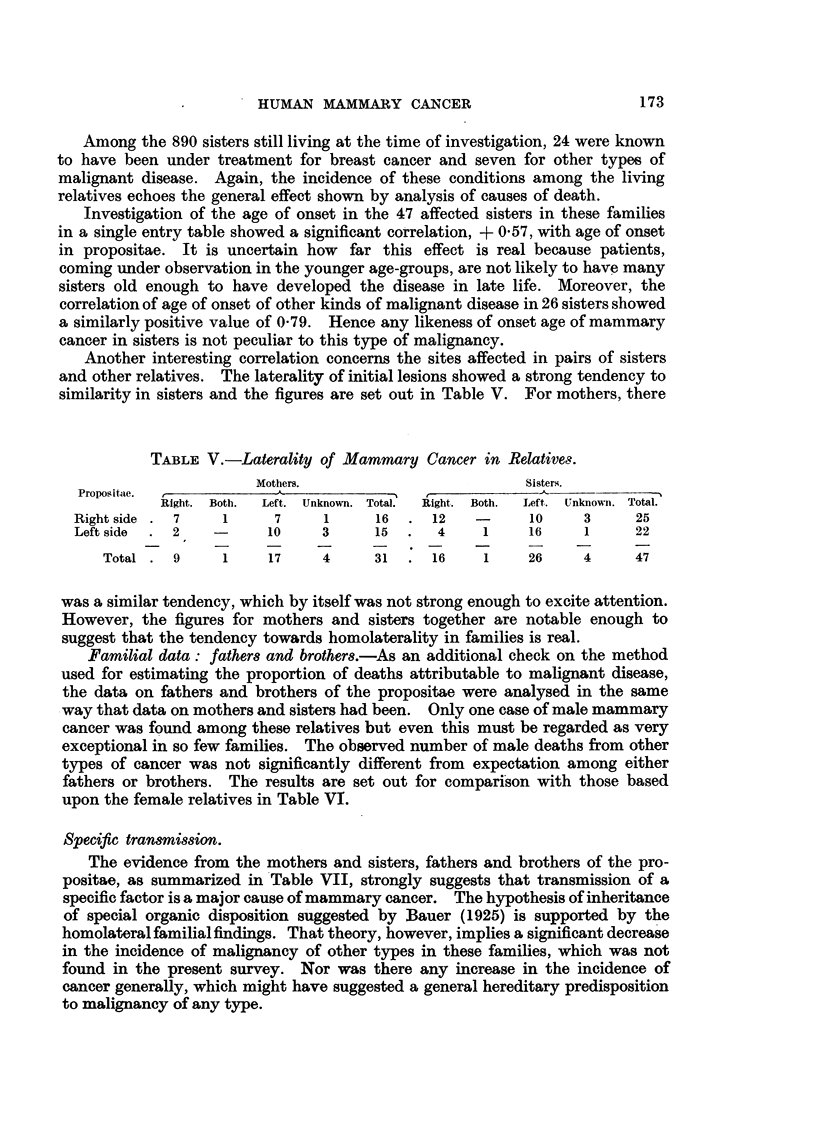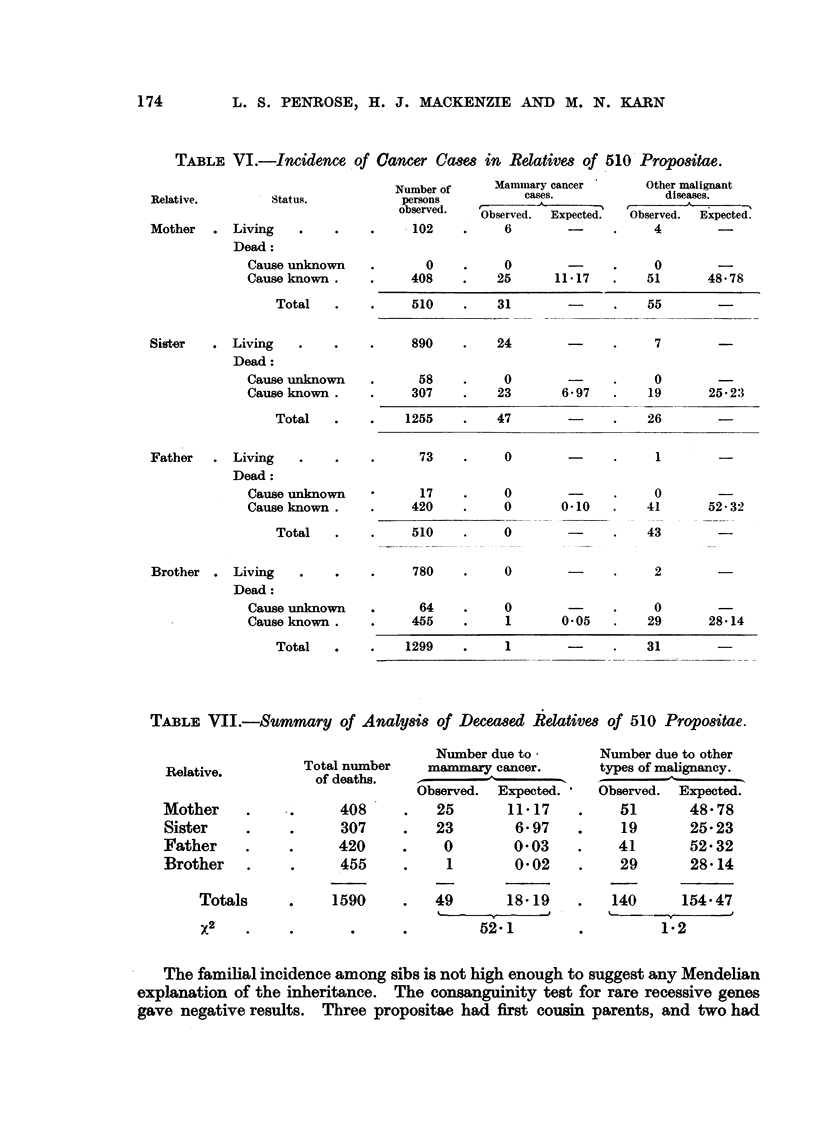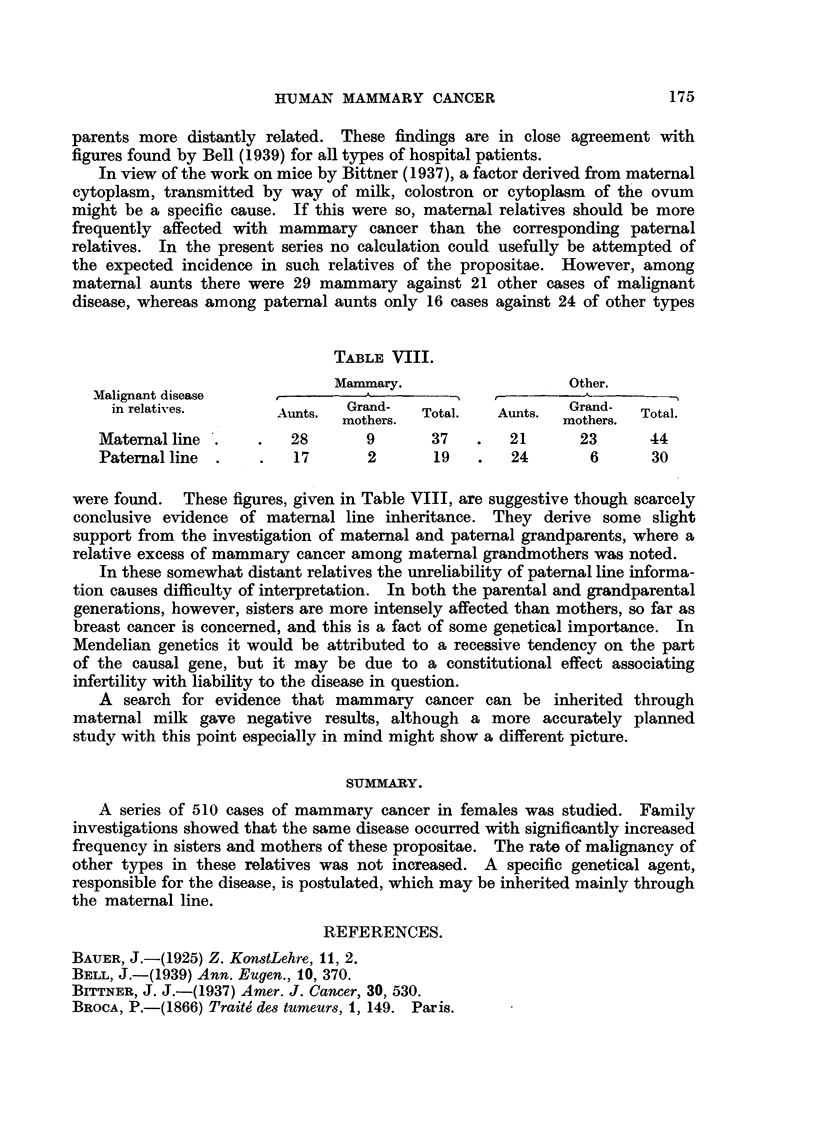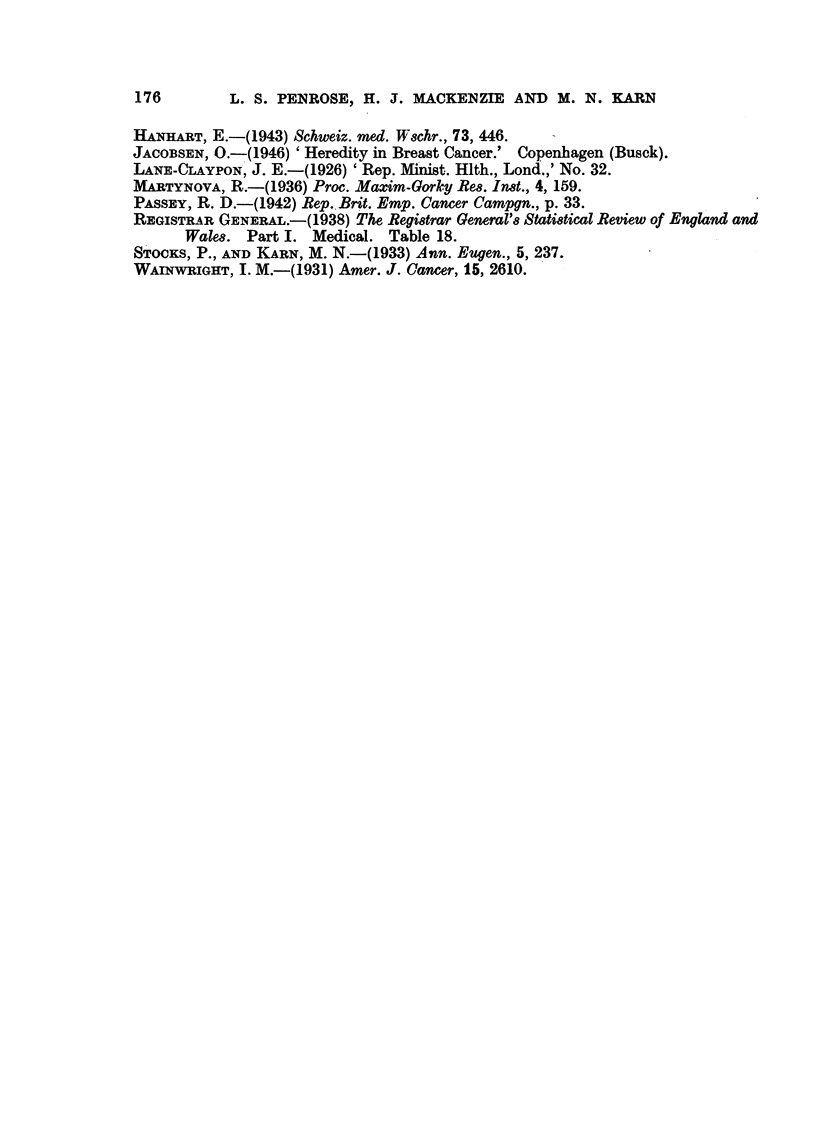# A Genetical Study of Human Mammary Cancer

**DOI:** 10.1038/bjc.1948.25

**Published:** 1948-06

**Authors:** L S Penrose, H. J. Mackenzie, M. N. Karn


### A GENETICAL STUDY OF HUMAN MAMMARY CANCER.

L S PENROSE, H. J. MACKENZIE AND M. N. KARN.

*From the Galton Laboratory, University College, London.*

Given at the Symposium on the Genetics of Cancer, London, June 24 and 25, 1948.

THE study of the mammary cancer forms a most advantageous starting-point for the investigation of the hereditary factors in cancers, because it is more easily recognized and more accurately diagnosed than malignant growths at most other sites. Moreover it produces, in the great majority of patients, a clearly defined range of pathological conditions, which include spheroidal-celled carcinoma, Paget's disease of the nipple, adeno-carcinoma, and the typical scirrhous condition in long-standing cases.

The history of investigations into the genetic aspect has been summarized by Jaçobsen (1946). Some important records of pedigrees, showing apparent transmission from mother to daughter, were published nearly a century ago. The most remarkable was recorded by Broca (1866), in which four generations of females had the disease.

In spite of evidence brought forward by a great many observers that mammary cancer can frequently be found affecting several of the female members of a family group, doubt still remains as to whether heredity plays any significant aetiological part. This doubt arises because of the great prevalence of the disease in the general population, where it is responsible for nearly 3 per cent of all female deaths. With so common a condition, it is difficult to demonstrate convincingly that the occasional familial concentration is not merely the result of a random distribution of cases.

To obviate this uncertainty, most investigators in recent years have collected a series of cases, noted the incidence of similar disease among their relatives, and compared it with the corresponding incidence among a control group of cases free from the disease, selected so far as possible at random. In spite of elaborate precautions, the difficulties, involved in obtaining a satisfactory control group of family histories to match those of the propositae, have repeatedly proved almost insuperable. The result is that many workers (Hanhart, 1943 ; Passey, 1942) still consider that there is insufficient evidence to prove the reality of familial transmission of mammary cancer or of any type of human malignant disease. Stocks and Karn (1933) found no evidence that a history of cancer in relatives increased the risk of cancer to any measurable extent.

Several extensive surveys have been published, all of which give some evidence for believing that mammary cancer is unusually prevalent among the mothers and sisters of affected cases. Among the important recent investigations are those of Lane-Claypon (1926) on 508 propositae, Wainwright (1931) on 784, Martynova (1936) on 201 and Jacobsen (1946) on 200 propositae. In all these investigations, however, some uncertainty remains as to whether the incidence of cancer cases, ascertained in the control populations, is correct. The carefully chosen control group examined by Jacobsen contained indeed far fewer cases than would be expected to occur on the hypothesis that the mortality within the group is the same as that of the general population. The result of these doubts is to reduce considerably the force of demonstrations which might otherwise seem very convincing.

### *The Present Investigation.*

The survey now described was undertaken with two main objectives. In the first place it was hoped that a direct comparison of deaths from different types of cancer in the relatives of a series of propositae could be compared directly with the rates given in statistics of the general population. This should remove some of the uncertainties inherent in collecting a control group for comparison, and make possible fairly accurate inferences about the existence or otherwise of familial concentration of cases. Secondly, the material was compiled in such a way that, if the result should show definite evidence for the hereditary transmission of mammary cancer, indications of the type of inheritance could also be obtained. In particular, the possibilities of transmission through maternal milk or the cytoplasm were considered as possible hypotheses. Inheritance of a factor causing special predisposition of certain sites in some families but not in others was kept in mind also as a possibility.

The material, which was collected, started in each instance with a patient attending a clinic after the diagnosis of mammary cancer had been made by a competent surgeon or pathologist and, often, by both. The patients were attending either University College Hospital or the London Hospital, and they were personally interviewed. All possible hospital records and reports were searched or checked by correspondence. This procedure applied both to the propositae and to any of their relatives who were reported to have suffered from malignant disease. Unfortunately, in a number of instances, records had been destroyed by enemy action during the war. Ideally, the death certificate of every relative believed to have died of malignant disease should have been obtained, as corroborative evidence, but this we have not been able to do. The routine investigation of family histories was confined to each patient's parents, brothers and sisters, children, grandparents, uncles and aunts. These data, it is hoped, may prove to be more valuable than those previously collected in that, with careful questioning, the year of death as well as the age was obtained. This information was complete for the mothers who had died, less so for the sibs and fathers.

Altogether 521 histories of cases of mammary cancer were collected. In nine of these the diagnosis was considered to be unreliable and they were rejected. There were, also, two male cases and these were set aside ; in neither was anything of note found in the family history. The 510 remaining cases were analysed from a number of different points of view.

#### *Analysis of the sample.*

The mean age at onset of the disease in the 510 cases was 51·7 years, with a standard deviation of 11·4 years. The distribution is set out in Table I.

TABLE I.—*Age of Onset of Disease in Propositae.*

| Age. | Total number. | Number with at least one affected relative. |
|---|---|---|
| 20– | 1 | — |
| 25– | 5 | 2 |
| 30– | 21 | 4 |
| 35– | 43 | 11 |
| 40– | 70 | 13 |
| 45– | 101 | 25 |
| 50– | 72 | 19 |
| 55– | 59 | 14 |
| 60– | 64 | 15 |
| 65– | 38 | 11 |
| 70– | 25 | 1 |
| 75– | 9 | 1 |
| 80– | 2 | — |
| 85– | — | — |
| All ages | 510 | 116 |
| Mean (years) | 51·7 | 51·0 |
| Standard deviation (years) | 11·4 | 10·4 |

In the pedigrees of 116 instances, one or more relatives with mammary cancer were ascertained. The distribution of these 116 "familial" cases and the mean age of onset of their illness (51·0 years) was extremely similar to that of the remaining "non-familial" cases. This finding does not support Jacobsen's assertion that familial cases have an earlier onset than others. Among the group of 510 women, 408 were married but 91 of these had no children. The infertility ratio, 91 out of 408, or 22·3 per cent, significantly exceeds the value of 17·3 per cent for married women of the same mean age given by the Registrar-General (1938), and supports the view that nulliparity is an etiological factor.

The mean age of the patients when first interviewed for purposes of this survey was 55·2 years. This indicates that they had, on the average, lived three-and-a-half years since first signs of the disease had appeared. Undoubtedly the survival of the patient was a property, which determined her inclusion in the investigation, and the longer the survival, the more probably would she be selected as a proposita. On the whole, therefore, there has been a slight, unavoidable tendency to deal with cases in whom treatment has been comparatively successful. Conversely, many of them were still under radiological treatment, and others were attending hospital on account of recurrence, so that some cases may have been inadvertently selected on account of undue severity of symptoms. These considerations must be kept in mind when interpreting the data, particularly when considering the distribution of cases with respect to age of onset.

Of the 510 propositae, 255 were affected first on the left side and 253 on the right side; in two instances there was uncertainty on this point.

*Familial data: mothers.*—The mothers of 408 patients were ascertained to have died and, in the case of 25 of these women, death was due to mammary cancer. Other types of malignant disease accounted for the deaths of 51 of the mothers of propositae. The date of death and the age at the time were recorded for each case. In a few instances the dates were approximate, but could hardly be more than a few years in error. The expected proportion of deaths due to mammary cancer and other types of malignancy in each year and at each age can be obtained on the basis of the general population statistics. An example of such results is shown in Table II.

TABLE II.—*Expected Proportion of Deaths Due to Cancer. Females, England and Wales,* 1925–29.

| Age group. | Other types of malignancy. | Mammary cancer. |
|---|---|---|
| 0–9 | 0·0023 | 0·0000 |
| 10–19 | 0·0115 | 0·0000 |
| 20–24 | 0·0143 | 0·0003 |
| 25–29 | 0·0243 | 0·0037 |
| 30–34 | 0·0543 | 0·0131 |
| 35–39 | 0·0903 | 0·0298 |
| 40–44 | 0·1409 | 0·0526 |
| 45–49 | 0·1754 | 0·0653 |
| 50–54 | 0·1938 | 0·0632 |
| 55–59 | 0·1917 | 0·0521 |
| 60–64 | 0·1807 | 0·0408 |
| 65–69 | 0·1613 | 0·0294 |
| 70–74 | 0·1384 | 0·0216 |
| 75–79 | 0·0979 | 0·0168 |
| 80 and over | 0·0533 | 0·0122 |
| All ages | 0·1243 | 0·0239 |

Thus the expected number of deaths from these two causes among the mothers of the propositae could be calculated. The distribution of deaths by year and age is shown in Table III.

From this table the number of deaths due to mammary cancer if the women had been subject to the mortality in the general population was estimated at 11·17, a figure significantly less than the observed 25. For other types of malignancy the mortality among mothers of propositae showed no significant increase above expected total, i.e. 51 observed as compared with 48·76 expected.

There were 102 mothers still living at the time of investigation and, of these, six were known to be under treatment for mammary cancer while four had some other type of malignant disease. No correlation between age of onset of breast cancer in mothers and age of onset in patients could be demonstrated.

*Familial data : sisters.*—A similar survey of the patients' sisters revealed that, out of a total of 1255 individuals, 365 had died, though the cause and date were unknown for 58 of these. For the remaining 307 deaths the ages and dates were classified as shown in Table IV. Calculating the expected number of deaths due to mammary cancer, as with the mothers, led to a value of 6·98.

TABLE III.—*Distribution of Deaths of Mothers of Propositae by Age and Date.*

| Age. | Date. '80— | '85— | '90— | '95— | '00— | '05— | '10— | '15— | '20— | '25— | '30— | '35— | '40— | '55— | All dates. | (A.) | (B.) |
|---|---|---|---|---|---|---|---|---|---|---|---|---|---|---|---|---|---|
| 20–24 | 1 | .. | .. | .. | .. | .. | .. | .. | .. | .. | .. | .. | .. | .. | 1 | — | — |
| 25–29 | .. | .. | .. | .. | 1 | .. | .. | .. | .. | .. | .. | .. | .. | .. | 1 | — | — |
| 30–34 | .. | 5 | 2 | 1 | 2 | 1 | 1 | .. | .. | .. | .. | .. | .. | .. | 12 | 1 | — |
| 35–39 | 1 | 3 | 2 | 1 | 3 | 2 | 1 | 2 | .. | .. | 1 | .. | .. | .. | 16 | — | — |
| 40–44 | .. | .. | 2 | 3 | 1 | 3 | 3 | 2 | .. | .. | .. | .. | .. | .. | 14 | 1 | 1 |
| 45–49 | .. | .. | 3 | .. | 3 | 2 | 2 | 2 | 1 | 1 | .. | 1 | .. | .. | 15 | — | 2 |
| 50–54 | .. | 1 | .. | 4 | 1 | 3 | 3 | 4 | 5 | 5 | 2 | .. | .. | .. | 28 | 7 | 4 |
| 55–59 | .. | 1 | .. | 1 | 1 | 3 | 3 | 3 | 3 | 5 | 2 | 2 | 2 | .. | 26 | 4 | 3 |
| 60–64 | .. | .. | .. | 1 | 2 | 6 | 12 | 3 | 1 | 9 | 4 | 10 | 6 | .. | 54 | 11 | 7 |
| 65–69 | .. | .. | .. | .. | 2 | .. | 5 | 5 | 4 | 10′ | 7 | 13 | 6 | 1 | 53 | 8 | 3 |
| 70–74 | .. | .. | .. | .. | .. | .. | 4 | 7 | 5 | 12 | 4 | 18 | 4 | 4 | 58 | 9 | 3 |
| 75–79 | .. | .. | .. | .. | .. | 2 | 3 | 5 | 8 | 10 | 3 | 7 | 11 | 3 | 52 | 4 | 2 |
| 80–84 | .. | .. | .. | .. | .. | .. | 1 | 2 | 2 | 5 | 6 | 7 | 6 | 4 | 33 | 4 | — |
| 85–89 | .. | .. | .. | .. | .. | .. | .. | 2 | 4 | 2 | 2 | 9 | 9 | 3 | 31 | 1 | — |
| 90–94 | .. | .. | .. | .. | .. | .. | .. | .. | .. | 2 | 7 | .. | 2 | 2 | 13 | 1 | — |
| 95–99 | .. | .. | .. | .. | .. | .. | .. | .. | .. | .. | 1 | .. | .. | .. | 1 | — | — |
| All ages | 2 | 10 | 9 | 11 | 16 | 22 | 38 | 37 | 33 | 61 | 39 | 67 | 46 | 17 | 408 | .. | .. |
| (A) | — | 1 | — | 1 | 1 | 2 | 5 | 7 | 2 | 7 | 5 | 17 | 3 | — | .. | 51 | .. |
| (B) | — | — | 1 | 1 | — | 3 | 2 | 4 | 1 | 4 | 1 | 3 | 4 | 1 | .. | .. | 25 |

(A) Due to types of malignancy other than mammary cancer.
(B) Due to mammary cancer.

TABLE IV.—*Distribution of Deaths of Sisters of Propositae by Age and Date.*

| Age. | Date. '75— | '80— | '85— | '90— | '95— | '00— | '05— | '10— | '15— | '20— | '25— | '30— | '35— | '40— | '45— | All dates. | (A.) | (B. |
|---|---|---|---|---|---|---|---|---|---|---|---|---|---|---|---|---|---|---|
| Infancy | 17 | 21 | 14 | 15 | 8 | 14 | 10 | 2 | 1 | 1 | .. | .. | .. | .. | .. | 103 | — | — |
| 10–14 | .. | 2 | 1 | 1 | 1 | 3 | .. | 3 | .. | .. | .. | .. | .. | .. | .. | 11 | — | — |
| 15–19 | .. | .. | .. | 1 | .. | 2 | 1 | 2 | 2 | .. | .. | .. | .. | .. | .. | 8 | — | — |
| 20–24 | .. | .. | .. | 4 | 2 | .. | 1 | 4 | 2 | .. | 1 | .. | .. | .. | .. | 14 | 1 | — |
| 25–29 | .. | .. | 1 | .. | .. | .. | .. | 2 | 2 | 2 | 3 | .. | .. | .. | .. | 10 | — | — |
| 30–34 | .. | .. | .. | .. | 1 | 2 | .. | 3 | 1 | 4 | 1 | 4 | 4 | 2 | .. | 22 | 2 | 2 |
| 35–39 | .. | .. | .. | .. | .. | .. | .. | 1 | .. | 2 | 2 | .. | 1 | .. | 1 | 7 | — | 1 |
| 40–44 | .. | .. | .. | .. | .. | .. | 1 | .. | 3 | 1 | 2 | .. | 1 | 2 | 1 | 11 | 2 | 1 |
| 45–49 | .. | .. | .. | .. | .. | .. | .. | 1 | 1 | 2 | 1 | 2 | 3 | 2 | .. | 12 | 2 | 3 |
| 50–54 | .. | .. | .. | .. | .. | .. | .. | .. | .. | 2 | 7 | 3 | 2 | 7 | 3 | 24 | 3 | 5 |
| 55–59 | .. | .. | .. | .. | .. | .. | .. | 1 | .. | .. | .. | 7 | 4 | 5 | 1 | 18 | 2 | 4 |
| 60–64 | .. | .. | .. | .. | .. | .. | .. | .. | .. | .. | .. | 4 | 5 | 8 | 7 | 24 | 5 | 5 |
| 65–69 | .. | .. | .. | .. | .. | .. | .. | .. | .. | .. | .. | 4 | 3 | 5 | 8 | 20 | 1 | 1 |
| 70–74 | .. | .. | .. | .. | .. | .. | .. | .. | .. | .. | .. | 2 | 5 | 5 | 3 | 15 | 1 | 1 |
| 75–79 | .. | .. | .. | .. | .. | .. | .. | .. | .. | .. | .. | .. | .. | 1 | 5 | 6 | — | — |
| 80–84 | .. | .. | .. | .. | .. | .. | .. | .. | .. | .. | .. | .. | .. | 2 | .. | 2 | — | — |
| All ages | 17 | 23 | 16 | 21 | 12 | 21 | 13 | 19 | 12 | 14 | 17 | 26 | 28 | 39 | 29 | 307 | .. | .. |
| (A) | .. | .. | .. | .. | .. | .. | .. | 1 | 1 | 1 | .. | 4 | 7 | 2 | 3 | .. | 19 | .. |
| (B) | .. | .. | .. | .. | .. | .. | .. | .. | .. | 1 | 1 | 5 | 5 | 10 | 1 | .. | .. | 23 |

(A) Due to types of malignancy other than mammary cancer.
(B) Due to mammary cancer.

Actually 23 had died of this condition. Only 19 had died of other types of malignant disease against an expected number of 25·21. Again, there is a highly significant increase in deaths from mammary cancer, even more marked than for mothers, but no significant divergence from expectation in respect of deaths from other types of malignancy.

Among the 890 sisters still living at the time of investigation, 24 were known to have been under treatment for breast cancer and seven for other types of malignant disease. Again, the incidence of these conditions among the living relatives echoes the general effect shown by analysis of causes of death.

Investigation of the age of onset in the 47 affected sisters in these families in a single entry table showed a significant correlation, + 0·57, with age of onset in propositae. It is uncertain how far this effect is real because patients, coming under observation in the younger age-groups, are not likely to have many sisters old enough to have developed the disease in late life. Moreover, the correlation of age of onset of other kinds of malignant disease in 26 sisters showed a similarly positive value of 0·79. Hence any likeness of onset age of mammary cancer in sisters is not peculiar to this type of malignancy.

Another interesting correlation concerns the sites affected in pairs of sisters and other relatives. The laterality of initial lesions showed a strong tendency to similarity in sisters and the figures are set out in Table V. For mothers, there

TABLE V.—*Laterality of Mammary Cancer in Relatives.*

| Propositae. | Mothers. | | | | | Sisters. | | | | |
|---|---|---|---|---|---|---|---|---|---|---|
| | Right. | Both. | Left. | Unknown. | Total. | Right. | Both. | Left. | Unknown. | Total. |
| Right side | 7 | 1 | 7 | 1 | 16 | 12 | — | 10 | 3 | 25 |
| Left side | 2 | — | 10 | 3 | 15 | 4 | 1 | 16 | 1 | 22 |
| Total | 9 | 1 | 17 | 4 | 31 | 16 | 1 | 26 | 4 | 47 |

was a similar tendency, which by itself was not strong enough to excite attention. However, the figures for mothers and sisters together are notable enough to suggest that the tendency towards homolaterality in families is real.

*Familial data : fathers and brothers.*—As an additional check on the method used for estimating the proportion of deaths attributable to malignant disease, the data on fathers and brothers of the propositae were analysed in the same way that data on mothers and sisters had been. Only one case of male mammary cancer was found among these relatives but even this must be regarded as very exceptional in so few families. The observed number of male deaths from other types of cancer was not significantly different from expectation among either fathers or brothers. The results are set out for comparison with those based upon the female relatives in Table VI.

*Specific transmission.*

The evidence from the mothers and sisters, fathers and brothers of the propositae, as summarized in Table VII, strongly suggests that transmission of a specific factor is a major cause of mammary cancer. The hypothesis of inheritance of special organic disposition suggested by Bauer (1925) is supported by the homolateral familial findings. That theory, however, implies a significant decrease in the incidence of malignancy of other types in these families, which was not found in the present survey. Nor was there any increase in the incidence of cancer generally, which might have suggested a general hereditary predisposition to malignancy of any type.

TABLE VI.—*Incidence of Cancer Cases in Relatives of* 510 *Propositae.*

| Relative. | Status. | Number of persons observed. | Mammary cancer cases. | | Other malignant diseases. | |
|---|---|---|---|---|---|---|
| | | | Observed. | Expected. | Observed. | Expected. |
| Mother | Living | 102 | 6 | — | 4 | — |
| | Dead: | | | | | |
| | Cause unknown | 0 | 0 | — | 0 | — |
| | Cause known | 408 | 25 | 11·17 | 51 | 48·78 |
| | Total | 510 | 31 | — | 55 | — |
| Sister | Living | 890 | 24 | — | 7 | — |
| | Dead: | | | | | |
| | Cause unknown | 58 | 0 | — | 0 | — |
| | Cause known | 307 | 23 | 6·97 | 19 | 25·23 |
| | Total | 1255 | 47 | — | 26 | — |
| Father | Living | 73 | 0 | — | 1 | — |
| | Dead: | | | | | |
| | Cause unknown | 17 | 0 | — | 0 | — |
| | Cause known | 420 | 0 | 0·10 | 41 | 52·32 |
| | Total | 510 | 0 | — | 43 | — |
| Brother | Living | 780 | 0 | — | 2 | — |
| | Dead: | | | | | |
| | Cause unknown | 64 | 0 | — | 0 | — |
| | Cause known | 455 | 1 | 0·05 | 29 | 28·14 |
| | Total | 1299 | 1 | — | 31 | — |

TABLE VII.—*Summary of Analysis of Deceased Relatives of* 510 *Propositae.*

| Relative. | Total number of deaths. | Number due to mammary cancer. | | Number due to other types of malignancy. | |
|---|---|---|---|---|---|
| | | Observed. | Expected. | Observed. | Expected. |
| Mother | 408 | 25 | 11·17 | 51 | 48·78 |
| Sister | 307 | 23 | 6·97 | 19 | 25·23 |
| Father | 420 | 0 | 0·03 | 41 | 52·32 |
| Brother | 455 | 1 | 0·02 | 29 | 28·14 |
| Totals | 1590 | 49 | 18·19 | 140 | 154·47 |
| $\chi^2$ | | 52·1 | | 1·2 | |

The familial incidence among sibs is not high enough to suggest any Mendelian explanation of the inheritance. The consanguinity test for rare recessive genes gave negative results. Three propositae had first cousin parents, and two had parents more distantly related. These findings are in close agreement with figures found by Bell (1939) for all types of hospital patients.

In view of the work on mice by Bittner (1937), a factor derived from maternal cytoplasm, transmitted by way of milk, colostron or cytoplasm of the ovum might be a specific cause. If this were so, maternal relatives should be more frequently affected with mammary cancer than the corresponding paternal relatives. In the present series no calculation could usefully be attempted of the expected incidence in such relatives of the propositae. However, among maternal aunts there were 29 mammary against 21 other cases of malignant disease, whereas among paternal aunts only 16 cases against 24 of other types

TABLE VIII.

| Malignant disease in relatives. | Mammary. | | | Other. | | |
|---|---|---|---|---|---|---|
| | Aunts. | Grand-mothers. | Total. | Aunts. | Grand-mothers. | Total. |
| Maternal line . . | 28 | 9 | 37 | 21 | 23 | 44 |
| Paternal line . . | 17 | 2 | 19 | 24 | 6 | 30 |

were found. These figures, given in Table VIII, are suggestive though scarcely conclusive evidence of maternal line inheritance. They derive some slight support from the investigation of maternal and paternal grandparents, where a relative excess of mammary cancer among maternal grandmothers was noted.

In these somewhat distant relatives the unreliability of paternal line information causes difficulty of interpretation. In both the parental and grandparental generations, however, sisters are more intensely affected than mothers, so far as breast cancer is concerned, and this is a fact of some genetical importance. In Mendelian genetics it would be attributed to a recessive tendency on the part of the causal gene, but it may be due to a constitutional effect associating infertility with liability to the disease in question.

A search for evidence that mammary cancer can be inherited through maternal milk gave negative results, although a more accurately planned study with this point especially in mind might show a different picture.

### SUMMARY.

A series of 510 cases of mammary cancer in females was studied. Family investigations showed that the same disease occurred with significantly increased frequency in sisters and mothers of these propositae. The rate of malignancy of other types in these relatives was not increased. A specific genetical agent, responsible for the disease, is postulated, which may be inherited mainly through the maternal line.

### REFERENCES.

BAUER, J.—(1925) *Z. KonstLehre*, **11**, 2.
BELL, J.—(1939) *Ann. Eugen.*, **10**, 370.
BITTNER, J. J.—(1937) *Amer. J. Cancer*, **30**, 530.
BROCA, P.—(1866) *Traité des tumeurs*, 1, 149. Paris.

HANHART, E.—(1943) *Schweiz. med. Wschr.*, **73**, 446.
JACOBSEN, O.—(1946) 'Heredity in Breast Cancer.' Copenhagen (Busck).
LANE-CLAYPON, J. E.—(1926) 'Rep. Minist. Hlth., Lond.,' No. 32.
MARTYNOVA, R.—(1936) *Proc. Maxim-Gorky Res. Inst.*, **4**, 159.
PASSEY, R. D.—(1942) *Rep. Brit. Emp. Cancer Campgn.*, p. 33.
REGISTRAR GENERAL.—(1938) *The Registrar General's Statistical Review of England and Wales.* Part I. Medical. Table 18.
STOCKS, P., AND KARN, M. N.—(1933) *Ann. Eugen.*, **5**, 237.
WAINWRIGHT, I. M.—(1931) *Amer. J. Cancer*, **15**, 2610.